# Dietary l-Tryptophan Supplementation Enhances the Intestinal Mucosal Barrier Function in Weaned Piglets: Implication of Tryptophan-Metabolizing Microbiota

**DOI:** 10.3390/ijms20010020

**Published:** 2018-12-21

**Authors:** Haiwei Liang, Zhaolai Dai, Jiao Kou, Kaiji Sun, Jingqing Chen, Ying Yang, Guoyao Wu, Zhenlong Wu

**Affiliations:** 1 Beijing Advanced Innovation Center for Food Nutrition and Human Health, China Agricultural University, Beijing 100193, China; lianghaiwei1988@hotmail.com; 2 State Key Laboratory of Animal Nutrition, College of Animal Science and Technology, China Agricultural University, Beijing 100193, China; daizhaolai@163.com (Z.D.); sckoujiao@163.com (J.K.); sunkaiji@hotmail.com (K.S.); CJQ9512@163.com (J.C.); cauvet@163.com (Y.Y.); g-wu@exchange.tamu.edu (G.W.); 3 Department of Animal Science, Texas A&M University, College Station, TX 77843, USA

**Keywords:** tryptophan, microbiota, mucosal defense, tight junction proteins, piglets

## Abstract

l-Tryptophan (Trp) is known to play an important role in the health of the large intestine. However, a role of dietary Trp in the small-intestinal mucosal barrier and microbiota remains poorly understood. The present study was conducted with weaned piglets to address this issue. Postweaning piglets were fed for 4 weeks a corn- and soybean meal-based diet supplemented with 0 (Control), 0.1, 0.2, or 0.4% Trp. The small-intestinal microbiota and serum amino acids were analyzed by bacterial 16S rRNA gene-based high-throughput sequencing methods and high-performance liquid chromatography, respectively. The mRNA levels for genes involved in host defense and the abundances of tight-junction proteins in jejunum and duodenum were measured by real time-PCR and Western blot techniques, respectively. The concentrations of Trp in the serum of Trp-supplemented piglets increased in a dose-dependent manner. Compared with the control group, dietary supplementation with 0.2–0.4% Trp reduced the abundances of *Clostridium sensu stricto* and *Streptococcus* in the jejunum, increased the abundances of *Lactobacillus* and *Clostridium XI* (two species of bacteria that can metabolize Trp) in the jejunum, and augmented the concentrations of secretory immunoglobulin A (sIgA) as well as mRNA levels for porcine β-defensins 2 and 3 in jejunal tissues. Moreover, dietary Trp supplementation activated the mammalian target of rapamycin signaling and increased the abundances of tight-junction proteins (zonula occludens (ZO)-1, ZO-3, and claudin-1) in jejunum and duodenum. We suggested that Trp-metabolizing bacteria in the small intestine of weaned pigs primarily mediated the beneficial effects of dietary Trp on its mucosal integrity, health, and function.

## 1. Introduction

The commensal microbiota in the gastrointestinal tract has a profound effect on the maintenance of the small intestine, protection against invasive pathogens, and the maturation of the immune system, thereby benefiting the health of the host [1,2,3]. Both genetic and environmental factors (including stress and dietary nutrients) contribute to the intestinal homeostasis in humans and animals by regulating flora colonization [4,5,6]. Consistently, perturbation of the intestinal ecosystem is associated with impaired mucosal barrier function and enhanced susceptibility to various intestinal diseases [7,8,9]. Specifically, weaning stress, an inevitable event for infants and piglets, reconstructs the microbial ecological community in the gastrointestinal tract [10,11]. Recent studies have emphasized that dietary nutrients are critical factors affecting intestinal mucosal barrier function by regulating the intestinal microbiota of the host and inhibiting the colonization of pathogenic bacteria [12]. The intestinal flora can actively utilize dietary amino acids for protein synthesis and can modify the amino acid profile in the plasma of the host [13]. Interestingly, dietary supplementation with amino acids influences the composition and diversity of the intestinal microbiota, thus improving intestinal function [14,15].

l-Tryptophan (Trp) is a nutritionally essential amino acid in all animals. Results of in vitro studies indicate that Trp enhances the expression of tight junction proteins and promotes protein synthesis in intestinal porcine epithelial cells [16]. Dietary Trp is metabolized by the intestinal microbiota, liver, brain, and activated immunocytes [17]. About 25% of dietary Trp is catabolized by the small intestine in the first pass in weaned piglets [18,19]. Our previous studies with enterocytes isolated from piglets show that intestinal epithelial cells do not degrade Trp [16,20], indicating that intestinal bacteria are primarily responsible for the use of dietary Trp by the small intestine.

Recent studies have reported the existence of Trp-metabolizing bacteria in the small intestine, including *Lactococcus lactis subsp. cremoris*, *L. lactis subsp. lactis*, *Lactobacillus plantarum*, *Bacteroides*, *Streptococcus thermophilus*, *Escherichia coli K-12*, *Morganella morganii*, *Klebsiella pneumoniae*, and *Hafnia alvei* [21,22]. These bacteria can convert Trp to serotonin, indole, tryptamine, skatole, and indole acetate [17,23]. The metabolites of Trp can modify intestinal microbial composition, microbial metabolism, and the host-microbiome interface [17]. Dietary Trp supplementation improves the growth of weanling piglets and regulates the composition of the microbiota in their hindgut [24]. However, it remains unknown whether the Trp-metabolizing bacteria in the small intestine may contribute to the mucosal barrier function in Trp-supplemented piglets.

The present study was conducted with weaned piglets to test the hypothesis that dietary Trp could enhance the intestinal mucosal barrier function by enhancing defensin expression, regulating the abundance of tight junction proteins, activating the mammalian target of rapamycin (mTOR) signaling pathway, and promoting the enrichment of bacteria that can utilize Trp in the small intestine. The pig was used because it is a useful animal model for studying human nutrition and metabolism [15]. These two species share similarities in anatomy, physiology, metabolism, and intestinal microbiome [25,26,27].

## 2. Results

### 2.1. Concentrations of Amino Acids (AAs) in Serum

Compared with the control group, the concentration of Trp in the serum of weaned pigs was increased (*p* < 0.05) in a dose-dependent manner in response to dietary supplementation with Trp. The concentrations of L-glutamate, L-asparagine, L-histidine, and taurine in serum were elevated (*p* < 0.05) by the supplementation with 0.2% Trp, as compared with the controls. However, dietary supplementation with 0.1% Trp had no effect on the serum concentrations of amino acids except for L-glutamate, L-asparagine, and Trp (Table 1).

### 2.2. Expression of Tight Junction Proteins in the Small Intestine

**Figure 1:**
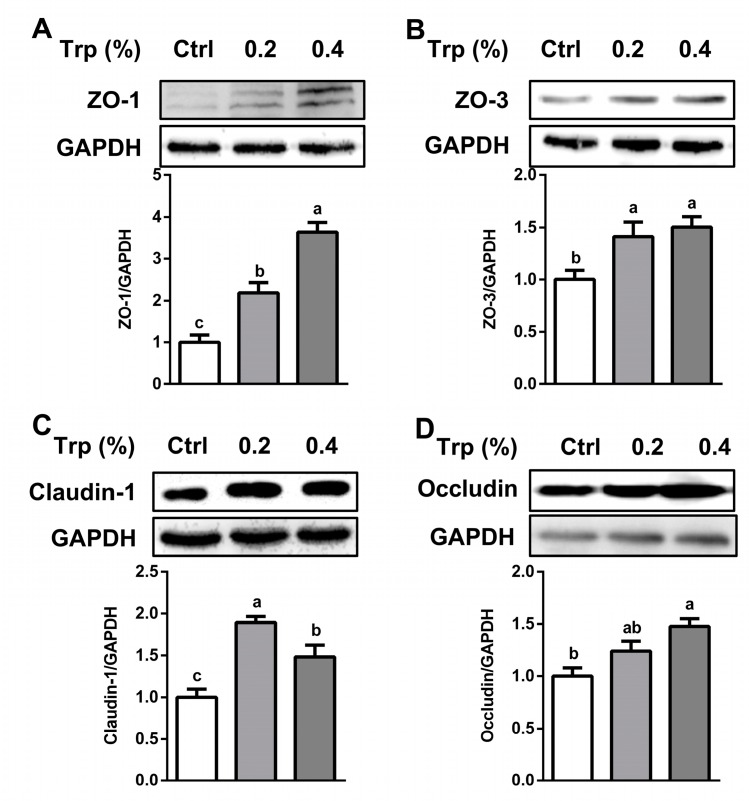
Protein abundances of ZO-1, ZO-3, claudin-1, and occludin in the jejunum of weaned piglets receiving dietary supplementation with 0% (Control, Ctrl), 0.2%, or 0.4% Trp. Western blot analyses of ZO-1 (**A**), ZO-3 (**B**), claudin-1 (**C**), and occludin (**D**) were performed. Values are means ± SEM, *n* = 6. Means without a common letter differ, *p* < 0.05.

Compared with the control group, the abundances of zonula occluden (ZO)-1, ZO-3, and claudin-1 proteins in the jejunum of weaned pigs were enhanced (*p* < 0.05) by dietary supplementation with both 0.2% and 0.4% Trp, whereas the protein abundance of occludin was elevated only by 0.4% Trp supplementation (*p* < 0.05) (Figure 1). The protein level of ZO-1 (Supplementary Figure S1A) in the duodenum was enhanced (*p* < 0.05) by 0.2% Trp, but not by 0.4% Trp. Additionally, the abundances of ZO-3 (Supplementary Figure S1B), claudin-1 (Supplementary Figure S1C), and occludin (Supplementary Figure S1D) were augmented (*p* < 0.05) by Trp supplementation in the duodenum.

### 2.3. Gene Expression of Porcine β-Defensin (pBD) and Secretory Immunoglobulin A (sIgA) Concentrations in the Jejunal Mucosa

**Figure 2:**
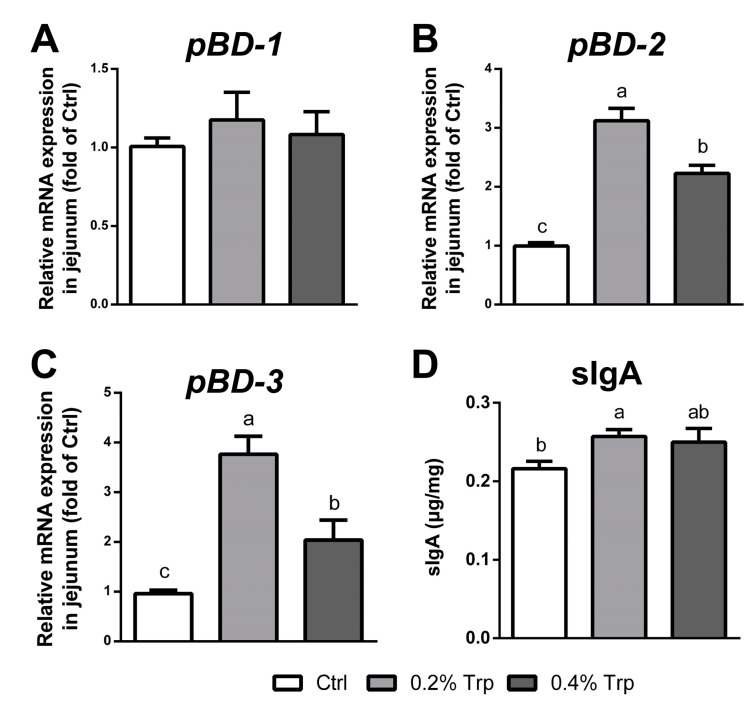
Dietary supplementation with Trp enhanced pBD expression and sIgA secretion in the small intestine of weaned piglets. (**A**–**C**) The mRNA levels of pBD-1, pBD-2, and pBD-3 in the mucosa of the jejunum. *n* = 6; and (**D**) the expression of sIgA in the mucosa of the jejunum. Values are means ± SEM, *n* = 6. Means without a common letter differ, *p* < 0.05. Ctrl, the control group (% Trp).

To explore the effect of dietary Trp supplementation on the mucosal defense, quantitative real-time PCR and ELISA assays were performed to determine mRNA levels of pBDs and the secretion of sIgA, respectively. Dietary supplementation with Trp (0.2%) increased (*p* < 0.05) the concentration of jejunal sIgA, whereas dietary supplementation with 0.2% or 0.4% Trp up-regulated (*p* < 0.05) jejunal pBD-2 and pBD-3 gene expression without affecting the pBD-1 gene expression (Figure 2).

### 2.4. The mTOR Signaling Pathway in the Small Intestine

**Figure 3:**
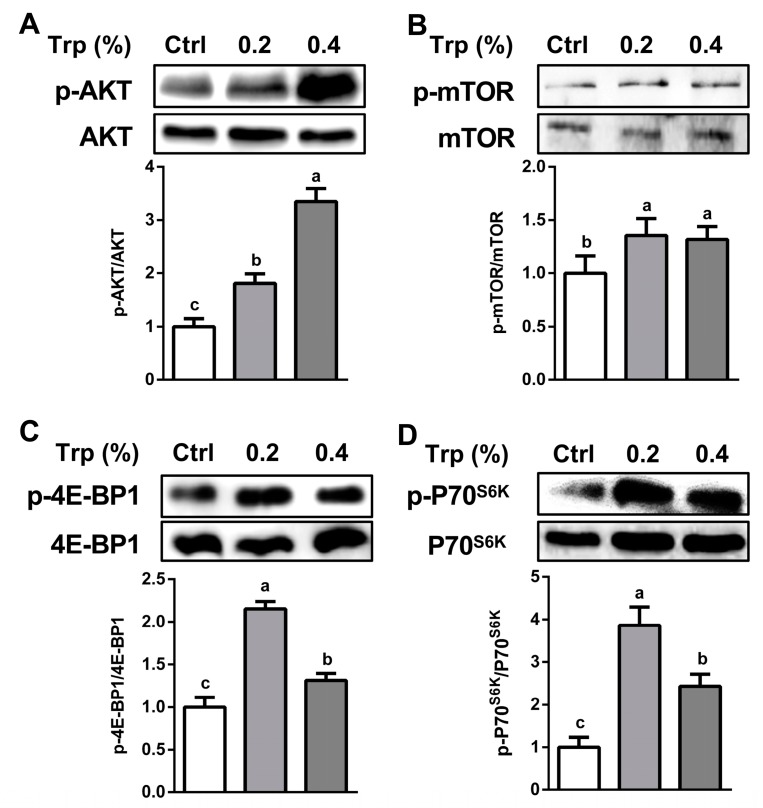
Dietary supplementation with Trp activated the mTOR signaling pathway in the jejunum of weaned piglets. Protein abundances of AKT (**A**), p-mTOR (**B**), p-4E-BP1 (**C**), and p-P70^S6K^ (**D**) were determined. Values are means ± SEM, *n* = 6. Means without a common letter differ, *p* < 0.05. Ctrl, the control group (% Trp).

**Figure 4:**
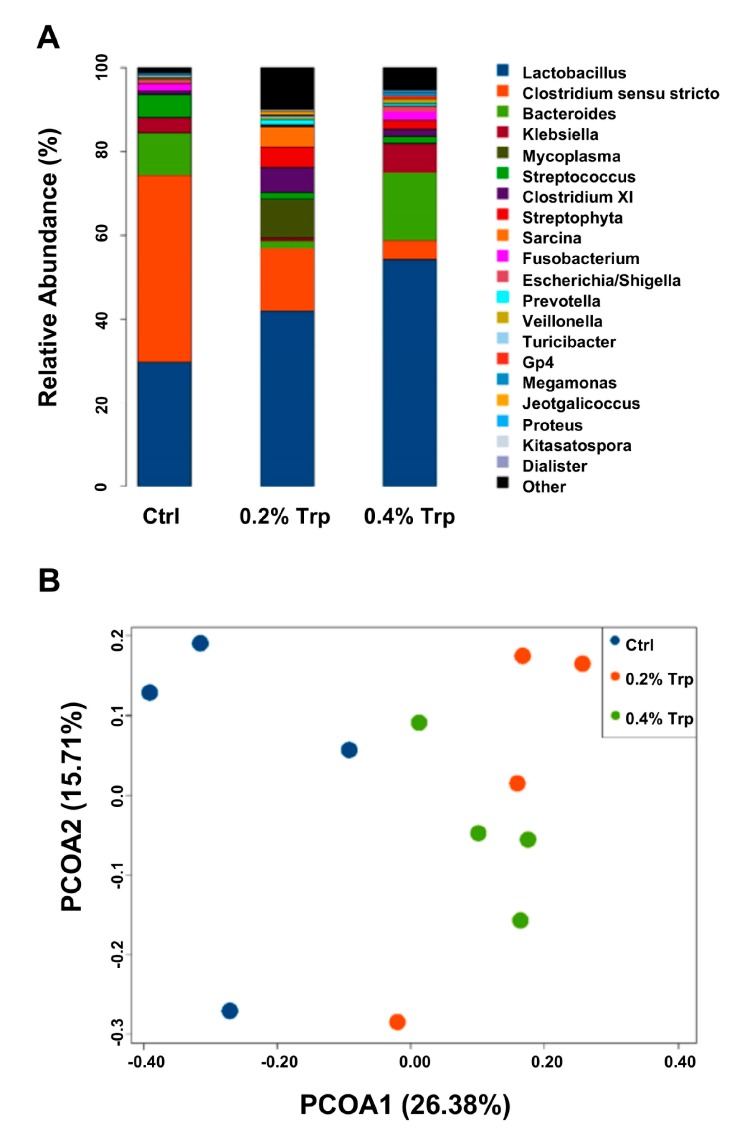
Microbiota composition and diversity in the jejunum of weaned piglets receiving dietary supplementation with Trp. (**A**) the composition of microbiota that was altered by Trp, and (**B**) the effect of Trp on the β diversity of the microbiota community. Ctrl, the control group (% Trp).

The abundance of protein kinase B (AKT) was enhanced (*p* < 0.05) by dietary Trp supplementation in both the jejunum and the duodenum (*p* < 0.05) (Figure 3A and Supplementary Figure S2A). In the jejunum, the abundances of p-mTOR (Figure 3B), p-4E (eIF4E)-binding protein 1 (4E-BP1) (Figure 3C), and p-p70 ribosomal protein S6 kinase (P70^S6K^) (Figure 3D) were enhanced (*p* < 0.05) by dietary supplementation with 0.2% or 0.4% Trp. In the duodenum, the abundances of p-mTOR (Supplementary Figure S2B) and p-P70^S6K^ (Supplementary Figure S2D) were enhanced (*p* < 0.05) by dietary supplementation with both 0.2% and 0.4% Trp, whereas the protein levels of total 4E-BP1 (Supplementary Figure S2C) and total ribosomal P70^S6K^ were not affected (*p* > 0.05) by dietary Trp.

### 2.5. Composition and Diversity of the Jejunal Microbiota

**Figure 5:**
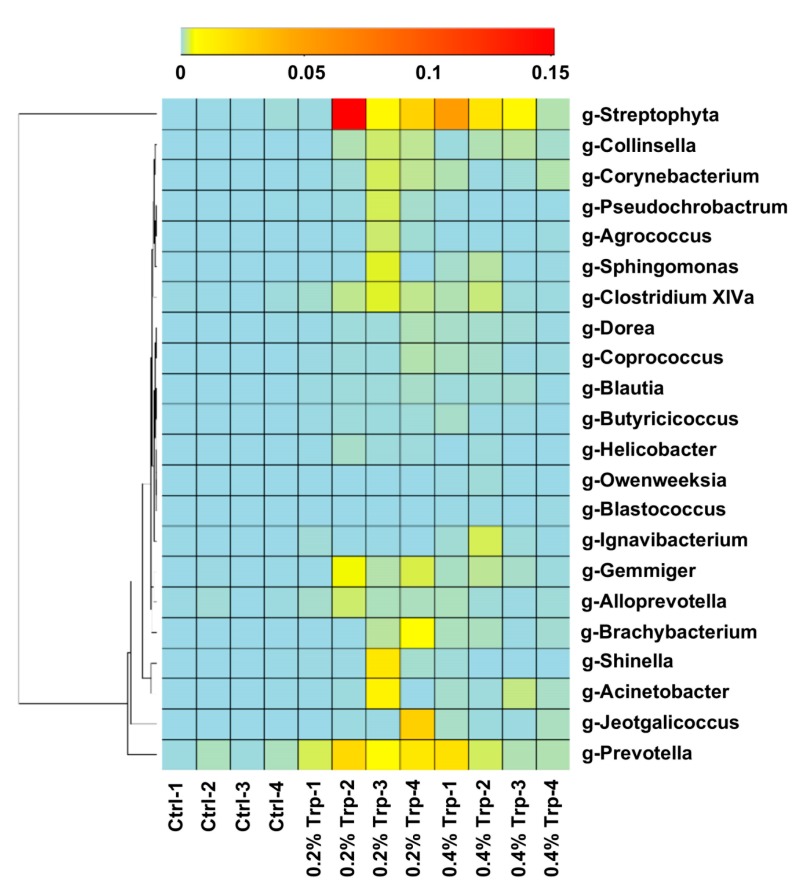
Heatmap cluster analysis of the bacteria at the genus level. The heatmap is color-coded based on row z-scores.

Dietary Trp supplementation altered (*p* < 0.05) the composition and diversity of the jejunal microbiota, especially the abundance of Trp-utilizing bacteria. After size filtering, quality control, and chimera checking, a total of 35,932 ± 921, 35,808 ± 943, and 30,309 ± 795 reads were observed in the 0%, 0.2%, and 0.4% Trp groups, respectively. Operational taxonomical units (OTUs) were obtained at a sequence-similarity level of 97%, and the number of OTUs in the 0.2% Trp group was higher than that in the control and 0.4% Trp groups. These alterations were accompanied with changes in Chao1 and observed species values (*p* < 0.05, Table 2). Furthermore, dietary Trp supplementation affected the abundance of Trp-metabolizing bacteria, such as an increase (*p* < 0.05) in the abundance of *Lactobacillus* and *Clostridium XI*, while reducing (*p* < 0.05) the abundance of opportunistic pathogens *Clostridium sensu stricto* and *Streptococcus* at the genus level (Figure 4A). The shaped bacterial composition at the phylum, class, order, and family levels are shown in Supplementary Figure S3. The principal coordinates analysis (PCoA) was used to assess β diversity among the three diet groups based on the unweighted unifrac distances, showing that the Trp supplementation groups formed a distinct cluster that was well separated from the control group along the first principal coordinates (Figure 4B). Based on the results of the heatmap, which can reflect the actual similarities and differences in the community composition of the samples, 22 genera in the jejunal microbiome were altered by dietary Trp supplementation (Figure 5).

## 3. Discussion

The intestinal microbiota has an important impact on key physiological functions in the host intestine, including metabolism, immune system maturation, and nutritional homeostasis [1,2,3]. Importantly, dietary factors have been reported to regulate the composition and diversity of the gut microbiota, therefore forming a crosstalk to modify the physiology and health of the host [28]. Despite a vast number of studies on the interaction between intestinal microbiota and host, the underlying mechanisms remain largely unknown. As a nutritionally essential amino acid, Trp has been reported to enhance intestinal protein synthesis and regulate the expression of tight junction proteins and intestinal transporters to benefit intestinal mucosal barrier function in humans and animals [16,22]. Of note, we found that intestinal porcine epithelial cells cannot degrade Trp [16,20], indicating a critical role of intestinal bacteria on Trp metabolism in the gastrointestinal tract. Our recent study showed that Trp supplementation could modulate the composition of microbiota in the hindgut of weaned piglets [24]. However, it is unknown whether the composition of bacteria in the small intestine, especially those that can metabolize Trp, can be regulated by Trp supplementation in weaned piglets.

In the present study, a corn- and soybean-based diet was formulated, according to nutritional requirements for weaned piglet. The basal diet contained 0.2% Trp and was supplemented with 0% (Control), 0.1%, 0.2%, or 0.4% Trp. We found that the concentrations of l-glutamate, l-asparagine, l-histidine, and taurine in serum were elevated in the 0.2% Trp group, as compared with the controls. It is possible that Trp modulates the intestinal absorption and whole-body metabolism of these amino acids in young piglets. However, the underlying mechanisms are currently unknown.

The intestinal mucosal barrier is maintained mainly by tight-junction proteins located between the epithelial cells [29]. We found that the abundances of ZO-1, ZO-3, occludin, and claudin-1 proteins in both the duodenum and the jejunum were upregulated by dietary supplementation with 0.2% or 0.4% Trp. This result is in agreement with our in vitro data from studies involving intestinal porcine epithelial cells [16] and a report that dietary Trp enhances the expression of tight-junction proteins in the intestine piglets infected by *Escherichia coli* [30]. Considering that the protein abundances of ZO-1, ZO-3, occludin, and claudin-1 were enhanced by Trp, it is imperative to determine whether Trp can exert such an effect in a bacteria-challenged model.

Host defense peptides exert both antimicrobial and immunomodulatory activities, and contribute to epithelial immune defense [31]. pBD is a major group of porcine antimicrobial peptides and plays an important role in both mucosal barrier function and immune response due to their antimicrobial, chemotactic, and regulatory activities [32,33]. Weaning stress and pathogen infection are associated with reduced expression of β-defensin [11,34]. Consistently, overexpression or exogenous supply of pBD improves the intestinal integrity and growth performance of weaned piglets [35]. Interestingly, we found that the mRNA levels of pBD-2 and pBD-3 were enhanced by Trp supplementation, indicating a regulatory effect of Trp on intestinal pBD gene expression. Furthermore, recent study has shown that the expression of defensins is tightly associated with the activation of mTOR cell signaling [36]. Consistently, we found that the expression of mTOR and its target genes in the 0.2% Trp group was higher than that in the 0.4% Trp group, indicating that an appropriate dosage of Trp was favorable for both the activation of mTOR cell signaling and the expression of the defensins. In addition to defensins, Trp supplementation also stimulated sIgA expression in the piglet small intestine. This protein plays an integral role in protecting the intestine against pathogen adherence by forming a mucus layer in the intestine [37,38]. These regulatory effects of Trp may be brought about through four mechanisms. First, Trp serves as a substrate for the intestinal synthesis of peptides and proteins, including pBD and sIgA [39]. Second, Trp regulates the expression of the pBD and sIgA genes through the Sirt1/ERK/90RSK signaling pathway [40]. Third, Trp can activate the mTOR cell signaling pathway in porcine enterocytes [16]. Fourth, the increased abundance of sIgA may be due to the altered microbiota composition following Trp supplementation, as the gut microbiota interacts directly or indirectly with the host immune system [12].

Intestinal microbiota dysfunction has been reported to be associated with impaired intestinal mucosal barrier and reduced growth performance in piglets [11]. In the present study, we found that the number of OTUs of the jejunal microbial flora and species richness (Chao1 and Observed species value) was increased by dietary Trp, indicating a regulatory effect on the intestinal microbiota in piglets. Several types of intestinal bacteria, such as *Lactococcus lactis subsp. cremoris*, *L. lactis subsp. lactis*, *Lactobacillus plantarum*, *Bacteroides*, *Streptococcus thermophilus*, *Escherichia coli*, *Morganella morganii*, *Klebsiella pneumoniae*, *Hafnia alvei,* and *Clostridium*, have tryptophanase for Trp catabolism [41] to maintain bacterial growth and survival [21,22,23,42,43,44]. Importantly, metabolites produced by these bacteria can regulate intestinal microbiota diversity and benefit the host [17]. In our study, the abundances of *Lactobacillus* and *Clostridium XI* in the small intestine were enhanced, whereas those of *Clostridium sensu stricto* and *Streptococcus* (two opportunistic pathogens of the intestine) were reduced by dietary Trp supplementation. *Lactobacillus* has been regarded as a beneficial intestinal bacterium for intestinal health in humans and animals [45], including weanling piglets [46]. An increase in the abundance of bacillius and a decrease in the abundances of opportunistic pathogens can contribute to an improved intestinal ecosystem. Of note, the regulatory effect of Trp on Trp-metabolizing bacteria was observed in the jejunum, instead of the hindgut [24], indicating a different response of different segment of the gastrointestinal tract to dietary Trp supplementation. The exact reason for this phenomenon remains unknown. It is possible that supplemental Trp does not enter the large intestine of pigs, because it is both absorbed into enterocytes and utilized by bacteria in the small intestine. It is also possible that the small intestine might be a suitable environment for the survival and colonization of Trp-metabolizing bacteria in piglets.

In conclusion, results of the present study indicate that dietary supplementation with Trp enhanced the intestinal mucosal barrier function as shown by the enhanced abundances of tight-junction proteins, as well as the upregulation of pBD and sIgA expression. These beneficial effects of Trp was associated with the activation of mTOR signaling and the enrichment of Trp-metabolizing bacteria in the small intestine of weaned pigs. Adequate provision of dietary Trp may be a nutritional strategy to improve intestinal mucosal barrier integrity, health and function in animals.

## 4. Materials and Methods

### 4.1. Experimental Design and Animals

All animal treatment and experimental procedures were approved by the China Agricultural University Animal Care Committee (No. CAU-DKY-20160308, 08 September 2016). A total of 168 crossbred weaned piglets (Landrace × Yorkshire) with similar bodyweights (7.6 kg of average BW, weaned at 24 d of age) were randomly assigned into one of the 4 groups: 0 (Control), 0.1, 0.2, and 0.4% supplemental Trp. l-Alanine was used to formulate isonitrogenous diets, as previously described [47]. Briefly, l-alanine is not toxic and can be extensively catabolized by pigs [48]. Additionally, in contrast to glycine and glutamate (substrate for the synthesis of glutamine and glutathione), which were confirmed as functional amino acids in the regulation of antioxidant function, l-alanine was not an antioxidant [49,50]. Additionally, dietary supplementation with glutamate, glycine, aspartate, and metabolites of tyrosine (dopa and dopamine) may alter food intake of pigs, due to their neuromodulator activity [51]. Furthermore, interconversion of biosynthesizable amino acids should not be ignored in animal nutrition [52]. For example, serine and asparagine are readily converted to glycine and aspartate in animals, respectively [53]. Therefore, among biosynthesizable amino acids, l-alanine is most appropriate for the isonitrogenous control. Each treatment group consisted of 6 pens (7 piglets/pen). The supplemental levels of Trp were based on a previous study showing that 0.23% supplemental Trp could increase the feed intake and body weight gain of piglets without any adverse effect [54]. The basal diet was formulated to meet nutritional requirements (NRC, 2012) of piglets throughout phase I (7–11 kg BW) (Supplementary Table S1) and phase II (11–25 kg BW) (Supplementary Table S2). In the present study, the day of weaning was recorded as day 0 of the experimental period. At the end of a four-week period of Trp supplementation, 24 piglets (8 from each of the 0, 0.2, and 0.4% Trp groups) were sacrificed by exsanguination. These three groups were chosen because dietary supplementation with 0.1% Trp had no effect on the growth performance of weaned piglets (Supplementary Table S3). Blood samples, the small intestinal tissues, and small intestinal contents were obtained from pigs in each treatment group. Serum, the small intestine, and intestinal contents were frozen in liquid nitrogen and then stored at –80 °C for later analysis.

### 4.2. Determination of Serum AAs by High-Performance Liquid Chromatography (HPLC)

The concentrations of AAs in serum were analyzed by HPLC methods as previously described [55], except that a model of Waters 2690 (Waters Chromatography Division, Milford, MA, USA) was used for separation and quantification.

### 4.3. Extraction of Proteins and Western Blot Analysis

Frozen duodenal and jejunal tissues were homogenized in liquid nitrogen for protein extraction and the analysis of protein abundance with the using of Western blot technique as previously described [56]. Briefly, 40 μg of protein was separated on 12% acrylamide SDS-PAGE gels, and then proteins were transferred onto PVDF membranes (Millipore, Billerica, MA, USA). The membranes were blocked with 5% skimmed-milk solution for 30 min at room temperature (25 °C), and then probed with a primary antibody overnight at 4 °C and subsequently incubated with an HRP-conjugated secondary antibody for 1 h at room temperature. The protein bands were detected with the Image Quant LAS 4000 mini system (GE Healthcare) and quantified with the use of the Quantity One software (Bio-Rad Laboratories, Hercules, CA, USA).

### 4.4. Quantitative Real-Time PCR Analysis

Total RNA was extracted from the tissues of jejunum using the Trizol reagent (Takara, Takara Biomedical Technology in Beijing. China), followed by reverse transcription using the High Capacity cDNA Archive kit (Takara), according to the manufacturer’s protocols. Real-time PCR was carried out by the ABI 7500 real-time PCR system (Applied Biosystems, Waltham, MA, USA) involving the use of SYBR Green. The primer sequences used for the mRNA determined were listed in Supplementary Table S4. The relative abundance of a target gene was calculated by the ΔΔ*C*t method [57].

### 4.5. Measurements for sIgA

The jejunal mucosa (0.1 g) were mixed with 0.1 mL physiological saline by tissue homogenate. Then sIgA was determined by using a porcine ELISA kit (Lianshuo Biochemical Reagent Company, Shanghai, China) according to the manufacturer’s instructions.

### 4.6. DNA Extraction and Bacterial 16S Ribosomal RNA (rRNA) Gene Sequencing

Jejunal bacteria were subjected to genomic DNA isolation with the use of the Qiagen DNA isolation kit (Qiagen, Hilden, Germany), according to the manufacturer’s instructions. The quality of isolated DNA was determined by agarose gel electrophoresis and then was stored at −20 °C until further processing. The 16S RNA V3-V4 gene region was amplified by using the primers F341 and R806 [58] and the 16S rRNA gene was sequenced on the Illumina HiSeq sequencing platform at the Realbio Genomics Institute (Shanghai, China). Sequences were quality filtered and clustered into OTUs at 97% identity [59].

### 4.7. Statistical Analysis

Data on serum AAs, α diversity indices (Chao1, Observed species, Shannon, and Simpson), protein abundances, and gene expression were analyzed by one-way ANOVA and the Duncan multiple comparison method (SPSS statistical software, SPSS Inc., Chicago, IL, USA). *p* < 0.05 was taken to indicate statistical significance. α diversity (Chao1, observed species, Shannon index, and Simpson index) was assessed by MOTHUR v.1.35.0 [60]. β diversity was calculated based on unweighted unifrac distances by QIIME. An unweighted unifrac PCoA based on OTUs was performed to provide an overview of the microbial diversity and composition in the pig jejunum.

## Figures and Tables

**Table 1:** The concentrations of amino acids in the serum of weaned piglets receiving dietary supplementation with l-tryptophan ^1^.

Item (nmol/mL)	Dietary Supplementation				SEM	*p*-Value
	0% Trp	0.1% Trp	0.2% Trp	0.4% Trp		
l-Asparate	49	50	56	52	1.69	0.536
l-Glutamate	144 ^b^	155 ^ab^	160 ^a^	156 ^ab^	2.62	0.035
l-Asparagine	80 ^b^	86 ^ab^	91 ^ab^	103 ^a^	3.79	0.036
l-Serine	196	198	214	204	5.03	0.608
l-Glutamine	540	540	571	558	10.3	0.692
l-Histidine	69 ^b^	70 ^b^	83 ^a^	76 ^ab^	2.30	0.031
Glycine	1139	1158	1220	1249	32.3	0.611
l-Threonine	114	121	122	135	3.71	0.254
l-Citruline	70	82	86	86	3.74	0.367
l-Arginine	222	230	234	259	11.6	0.731
Taurine	178 ^b^	212 ^b^	252 ^a^	231 ^ab^	9.18	0.020
l-Alanine	672	636	643	585	15.8	0.276
l-Tyrosine	124	127	129	139	5.42	0.812
l-Tryptophan	36 ^d^	52 ^c^	85 ^b^	105 ^a^	5.95	0.001
l-Methionine	41	43	42	47	2.01	0.737
l-Valine	200	201	217	241	7.89	0.218
l-Phenylalanine	100	89	100	100	2.66	0.350
l-Isoleucine	126	126	132	142	3.74	0.387
l-Leucine	215	214	216	234	6.06	0.620
l-Ornithine	116	123	141	143	5.34	0.192
l-Lysine	133	136	153	146	8.05	0.815

^1^ Values are means with the pooled SEM, *n* = 6. ^a, b, c, d^ Within a row, means not sharing the same superscript letter differ, *p* < 0.05.

**Table 2:** Effects of dietary supplementation with Trp on the α diversity indices (Chao1, Observed species, Shannon, and Simpson) and OTUs ^1^.

Items	Dietary Supplementation			SEM	*p*-Value
	0% Trp	0.2% Trp	0.4% Trp		
Chao1	127 ^b^	373 ^a^	304 ^a^	39.0	0.009
Observed species	87 ^c^	307 ^a^	242 ^b^	34.5	0.009
Shannon	2.03	3.56	3.34	0.39	0.240
Simpson	0.55	0.74	0.73	0.07	0.459
OTUs	110 ^c^	349 ^a^	276 ^b^	35.5	0.003

^1^ Values are means with the pooled SEM. ^a, b, c^ Within a row, means not sharing the same superscript letter differ, *p* < 0.05.

## Supplementary Materials

Supplementary materials can be found at http://www.mdpi.com/1422-0067/20/1/20/s1.

## Abbreviations

AAs	Amino acids
AKT	Protein kinase B
4E-BP1	4E (eIF4E)-binding protein 1
HPLC	High-performance liquid chromatography
mTOR	Mammalian (or mechanistic) target of rapamycin
OTUs	Operational taxonomical units
pBD	Porcine β-defensin
PCoA	Principal coordinates analysis
p70^S6K^	p70 ribosomal protein S6 kinase
sIgA	Secretory immunoglobulin A
Trp	l-Tryptophan
ZO	Zonula occluden
