# Supplementary material for: Dietary l-Tryptophan Supplementation Enhances the Intestinal Mucosal Barrier Function in Weaned Piglets: Implication of Tryptophan-Metabolizing Microbiota
Source: Int J Mol Sci. 2018 Dec 21;20(1):20. doi: 10.3390/ijms20010020 (PMC6337174; doi:10.3390/ijms20010020)
Supplement: Supplementary file 1 [file ijms-20-00020-s001.pdf]

Online Supporting Material

Supplementary Table S1. Ingredients and chemical composition of experimental diets (day 0-14). The basal diets of phase I (7-11 kg BW of piglets). BW: Body weight; DE: Digestible energy; CP: Crude protein.

Items	Diets			
	Ctrl	0.1% Trp	0.2% Trp	0.4% Trp
Ingredients%				
Corn	46.2	46.2	46.2	46.2
Extruded corn	12	12	12	12
Soybean meal (46%)	15.31	15.31	15.31	15.31
Extruded-soybean	10	10	10	10
Fish meal (63.0%)	4	4	4	4
Whey powder	6.5	6.5	6.5	6.5
Cornstarch	0.064	0.052	0.039	0.013
Soybean oil	2	2	2	2
L-Lysine (99.0%)	0.15	0.15	0.15	0.15
Trp (98.0%)	0	0.102	0.204	0.408
L-Alanine (99.8%)	0.446	0.356	0.267	0.089
Limestone powder	0.81	0.81	0.81	0.81
Dicalcium phosphate	1.12	1.12	1.12	1.12
Salt	0.4	0.4	0.4	0.4
1% Premix	1	1	1	1
Total	100	100	100	100
Nutrient levels % (Theoretical value)				
DE (Mcal/kg)	3.52	3.52	3.52	3.52
CP	19.49	19.49	19.49	19.49
L-Lysine	1.18	1.18	1.18	1.18
L-Methionine + L-Cysteine	0.68	0.68	0.68	0.68
L-Threonine	0.74	0.74	0.74	0.74
Trp	0.25	0.35	0.44	0.64
L-Isoleucine	0.75	0.75	0.75	0.75
Nutrient levels % (Chemical analysis)				
Total energy (Mcal/kg)	4.05	4.08	4.02	4.02
CP	20.62	21.05	20.12	20.15
Crude ash	5.3	5.3	5.8	5.3

Online Supporting Material

Supplementary Table S2. Ingredients and chemical composition of experimental diets (day 15-28). The basal diets of phase II (11-25 kg BW of piglets).

Items	Diets			
	Ctrl	0.1% Trp	0.2% Trp	0.4% Trp
Ingredients%				
Corn	55.25	55.25	55.25	55.25
Extruded-corn	12	12	12	12
Soybean meal (46%)	13.51	13.51	13.51	13.51
Extruded-soybean	10	10	10	10
Fish meal (63.0%)	3.28	3.28	3.28	3.28
Cornstarch	0.064	0.052	0.039	0.013
Soybean oil	2	2	2	2
L-Lysine (99.0%)	0.15	0.15	0.15	0.15
Trp (98.0%)	0	0.102	0.204	0.408
L-Alanine (99.8%)	0.446	0.356	0.267	0.089
Limestone powder	0.74	0.74	0.74	0.74
Dicalcium phosphate	1.17	1.17	1.17	1.17
Salt	0.4	0.4	0.4	0.4
1% Premix	1	1	1	1
Total	100	100	100	100
Nutrient levels % (Theoretical value)				
DE (Mcal/kg)	3.51	3.51	3.51	3.51
CP	18.41	18.41	18.41	18.41
L-Lysine	1.07	1.07	1.07	1.07
L-Methionine + L-Cysteine	0.65	0.65	0.65	0.65
L-Threonine	0.68	0.68	0.68	0.68
Trp	0.21	0.31	0.41	0.6
L-Isoleucine	0.69	0.69	0.69	0.69
Nutrient levels % (Chemical analysis)				
Total energy (Mcal/kg)	4.05	4.03	4.04	4.05
CP	18.64	18.93	18.63	18.62
Crude ash	4.5	5.4	4.8	4.7

Online Supporting Material

Supplementary Table S3. Effects of dietary Trp supplementation on the growth performance of weanling piglets. Piglets were weaned at 24 days of age and the first day of weaned was recorded as day 0. ADG: Average daily gain; ADFI: Average daily feed intake; FCR: Feed conversion ratio. Means without a common letter differ, $P < 0.05$.

Item	Diets				SEM	*P*-value
	Ctrl	0.1% Trp	0.2% Trp	0.4% Trp		
Initial BW, kg	7.67	7.66	7.61	7.69	0.03	0.876
Day14 BW, kg	10.9[b]	11[b]	11.3[a]	11.3[a]	0.06	0.011
Day28 BW, kg	17.6[b]	17.8[b]	18.8[a]	18.3[ab]	0.14	0.019
ADG, g						
d 0 to 14	228[b]	237[b]	264[a]	260[a]	4.45	0.003
d 15 to 28	484[b]	489[ab]	533[a]	496[ab]	7.89	0.026
d 0 to 28	356[b]	363[b]	398[a]	378[ab]	5.36	0.015
ADFI, g						
d 0 to 14	352[b]	355[b]	387[a]	382[a]	5.14	0.018
d 15 to 28	888	889	894	889	10.8	0.998
d 0 to 28	620	622	640	635	6.43	0.643
FCR						
d 0 to 14	1.55	1.50	1.47	1.47	0.01	0.211
d 15 to 28	1.84	1.83	1.69	1.79	0.03	0.339
d 0 to 28	1.74	1.72	1.61	1.68	0.02	0.213

Online Supporting Material

Supplementary Table S4: Primers sequences used for PCR.

Gene	Primer sequence (5'- 3')
pBD-1-F	TTCCTCCTCATGGTCCTGTT
pBD-1-R	AGGTGCCGATCTGTTTCATC
pBD-2-F	TGTCTGCCTCCTCTCTTCC
pBD-2-R	AACAGGTCCCTTCAATCCTG
pBD-3-F	CCTTCTCTTTGCCTTGCTCTT
pBD-3-R	GCCACTCACAGAACAGCTACC
GAPDH-F	CTCGGAGTGAACGGATTTGG
GAPDH-R	AGTGGAGGTCAATGAAGGGG
341-F	CCTACGGGRSGCAGCAG
806-R	GGACTACVVGGGTATCTAATC

Online Supporting Material

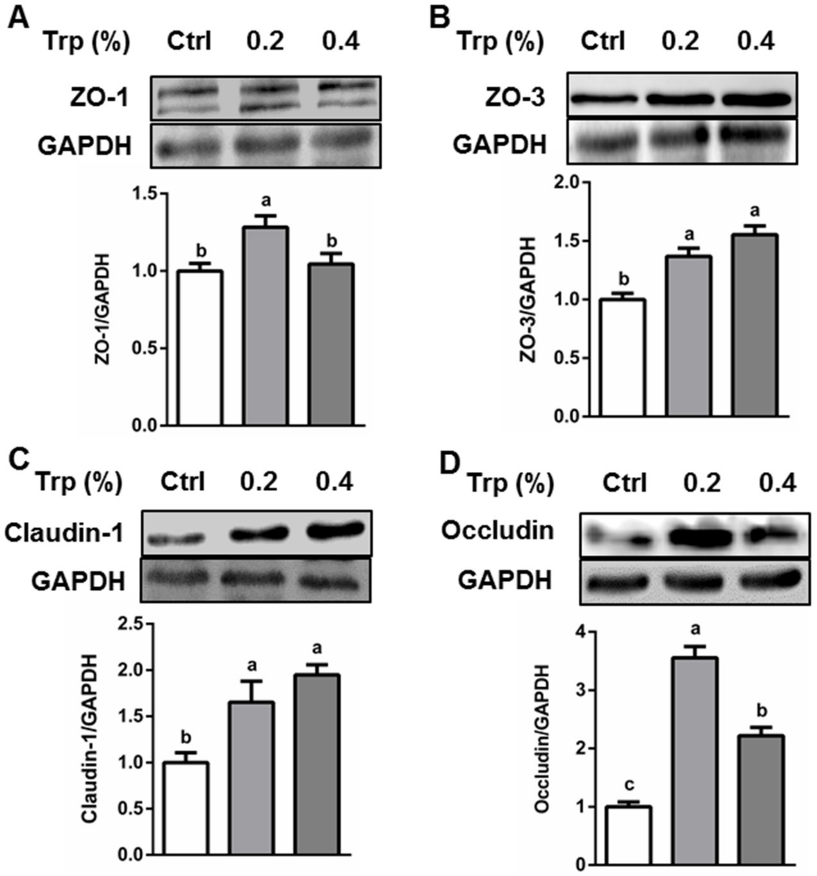

Supplemental Figure S 1. Protein abundance of ZO-1, ZO-3, Claudin-1 and Occludin in duodenum. Protein grayscale of ZO-1 (A), ZO-3 (B), claudin-1 (C) and occludin (D) were determined. Values are means ± SEMs, n = 6. Means without a common letter differ, $P < 0.05$.

Online Supporting Material

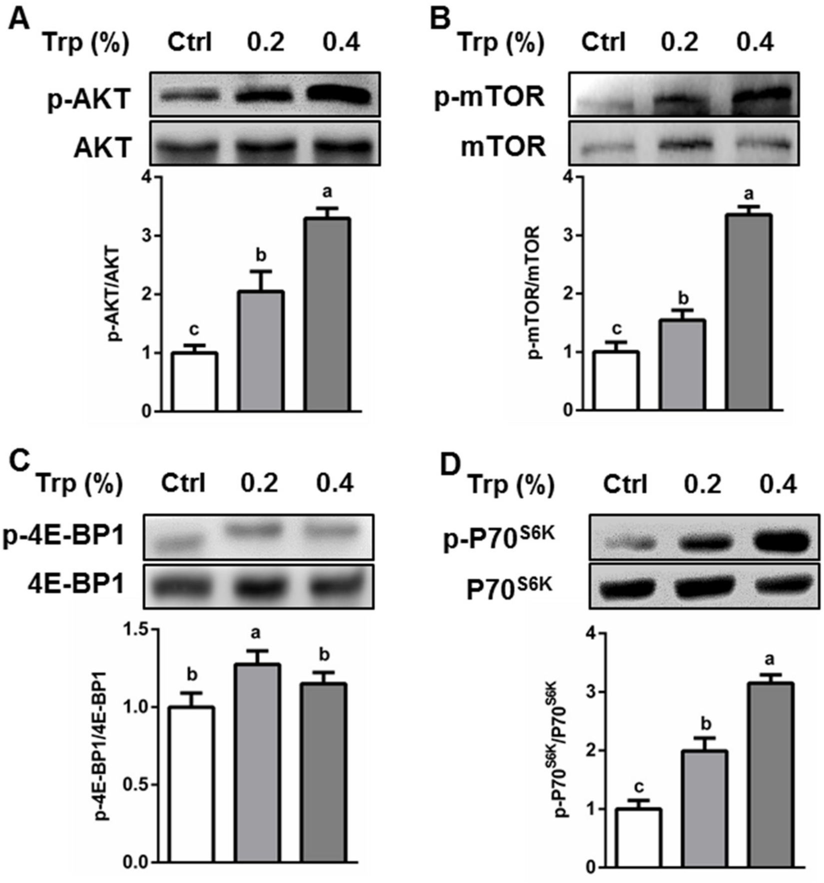

Supplemental Figure S2. Dietary Trp supplementation activates mTOR signaling pathway in duodenum. Protein grayscale of AKT (A), p-mTOR (B), p-4E-BP1 (C) and p-P70^{S6K} (D) were determined. Values are means ± SEMs, n = 6. Means without a common letter differ, $P < 0.05$.

Online Supporting Material

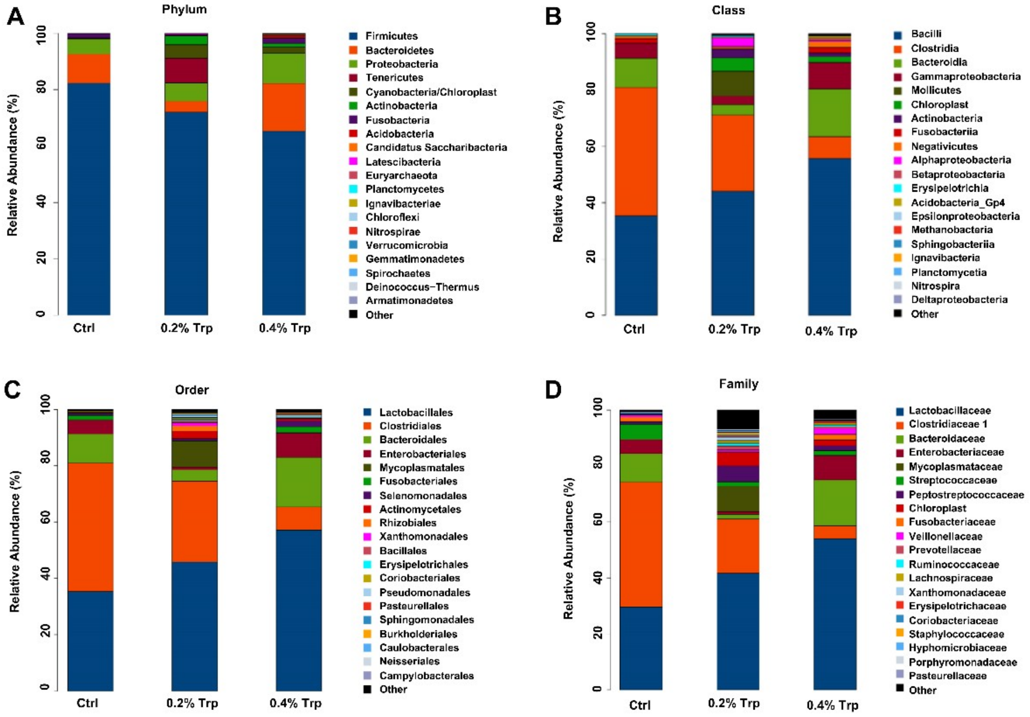

Supplemental Figure S3. Shaped bacteria composition at the phylum, class, order and family levels.
